# Advancing stroke diagnosis and management through nuclear medicine: a systematic review of clinical trials

**DOI:** 10.3389/fmed.2024.1425965

**Published:** 2024-08-19

**Authors:** Hala F. Azhari

**Affiliations:** College of Medicine and Pharmacy, Umm Al-Qura University, Makkah, Saudi Arabia

**Keywords:** ischemic stroke, hemorrhagic stroke, nuclear medicine, diagnosis, management, outcome prediction

## Abstract

**Introduction:**

Despite advancements in stroke care, challenges persist in timely triage and treatment initiation to prevent the burden of stroke-related disabilities. Although nuclear medicine has shown promise, no imaging technique has yet provided a sufficiently rapid, precise, and cost-effective approach to routine stroke management. This study aims to review the clinical application of nuclear medicine in stroke diagnosis and treatment.

**Methods:**

A systematic search of the Cochrane, EU Clinical Trials Register, ISRCTN, the International Stroke Trial, and the ClinicalTrials.gov database was conducted to find all registered trials reporting nuclear medicine’s clinical applications in stroke up to June 07, 2024.

**Results:**

Among the 220 screened trials, 51 (36 interventional; 15 observational) met the eligibility criteria. Participants were older than 18 years old, with only six studies including pediatric under 17 years old, with a total of 11,262 stroke (9,232 ischemic; 2,030 haemorrhagic) participants. The bias risk varied across trials but remained mostly low to moderate.

**Discussion:**

The review highlighted nuclear medicine’s significant contributions to stroke diagnosis and management, notably through mobile stroke units, pre-hospital acute stroke magnetic resonance image (MRI) based biomarkers, and MRI-based stroke mechanisms for 4D flow nuclear imaging. These advancements have generally reduced treatment delays and enhance clinical outcomes post-stroke. Specifically, radiopharmaceutical radiotracers can effectively discriminate between strokes and mimics, particularly in high-risk patients. Integrating novel positron emission tomography (PET) radiotracer 18F glycoprotein 1 and radionuclide angiography may improve sensitivity and specificity in thrombi detection for decisions regarding stenting or carotid endarterectomy, and the single-photon emission computed tomography and PET integration with ferumoxytol radiotracer-enhanced MRI enables functional imaging for evaluating cerebral perfusion, metabolic activity, and neuroinflammatory markers post-stroke. Overall, the integration of nuclear medicine into multimodal imaging equipment like computed-tomography PET and MRI-PET offers a more comprehensive picture of the brain. Nevertheless, further research is needed on novel stroke imaging techniques and standardization across stroke centers for optimal performance.

**Systematic review registration:**

https://www.crd.york.ac.uk/prospero/display_record.php?ID=CRD42024541680, identifier PROSPERO CRD(42024541680).

## 1 Introduction

Stroke is a major cause of morbidity and mortality, accounting for 30% of stroke-related disabilities and 20% of all deaths worldwide (1). Despite stroke system-optimization efforts, interhospital communication for triage and transport to a thrombectomy center can be operationally challenging. Even in experienced stroke care systems, there is a frequent time delay of nearly 100 min (2), which may result in significant neuronal loss, increased mortality, morbidity, or even exclusion as a candidate for endovascular thrombectomy (EVT) intervention. It is estimated that over 1.9 million neuronal fibers are lost each minute without treatment (3). Indeed, for every 15 min reduction to recanalization, 34 per 1,000 treated stroke patients experience improvements in disability, as faster initiation of treatment leads to better clinical outcomes (4).

The management of stroke patients depends highly on the information obtained from imaging technologies. Nuclear medicine utilizes radiopharmaceuticals to diagnose and manage strokes by emitting gamma rays detected by specialized cameras, which provide crucial images of the brain’s internal structures and functions (5). Technologies such as the single-photon emission computed tomography (SPECT) and positron emission tomography (PET) (6) offer molecular insights into disease processes and cerebral blood flow, thereby enabling personalized care. Despite the low radiation doses, the benefits of accurate diagnosis and targeted therapy, which lead to improved outcomes and stroke care quality, outweigh the associated risks.

Specifically, nuclear medicine techniques (7) of high sensitivity and specificity enable earlier diagnosis and more adequate management over computed-tomography (CT) and magnetic resonance image (MRI) scans. Determining stroke etiology, which remains cryptogenic in as many as one-third of patients even after a comprehensive workup (8), is challenging. This is in part because it is challenging not only to confidently identify stroke mimics, a false-positive stroke which explain more than 40% of cases presenting with an acute neurological deficit (9), but also to distinguish between heterogeneous ischemic and haemorrhagic stroke disorders in circumstances where access to brain imaging is limited and a very rapid turnaround is needed. Stroke treatment algorithms are becoming increasingly complex in addressing various clinical and biological parameters, imaging the infarct penumbra volume size, determining the time of symptom onset, and assessing haemorrhagic risk transformation. At present, the pathophysiological processes involved in brain damage and repair in the context of human stroke remain poorly understood, and successful adjunctive therapies remain limited.

The aim of this study was to review the existing evidence that demonstrates the promising translation of nuclear medicine into the clinical practice of stroke diagnosis and management and to describe newly reported clinical studies that could facilitate routine stroke care. Ultimately, this study provides valuable insights into the clinical applications of nuclear medicine in stroke care.

## 2 Materials and methods

The review followed the Preferred Reporting Items for Systematic Reviews guidelines (10). The study protocol was registered at the University of York, with a regeneration number PROSPERO CRD (42024541680) (11).

### 2.1 Data sources and search strategy

The Cochrane library, the EU Clinical Trials Register, the ISRCTN, the International Stroke Trial, and the ClinicalTrials.gov (12) were screened on June 07, 2024 to retrieve all eligible clinical trials. These databased served as a comprehensive online database registry of clinical trials worldwide. Within the database search engine filters, the search terms included keywords and synonyms associated with stroke as a ‘condition/disease’ and were conjugated with nuclear medicine as an ‘interventional’ option to identify all relevant results.

### 2.2 Inclusion and exclusion criteria

Interventional and non-interventional clinical trials qualified for inclusion if they predominantly focused on the clinical application of nuclear medicine for stroke diagnosis and/or treatment. Trials not in English language or not related to nuclear medicine use in stroke were excluded.

### 2.3 Data extraction

The pertinent data were manually extracted and downloaded from reports on the clinical databases website, based on pre-established eligibility criteria. A summary step gathered the key characteristics of each clinical trial, including study identification number, title, type of trial, stroke outcomes, interventions, nuclear imaging technology, primary and secondary outcome measures, sample size, age of participants enrolled, trial design, phase, and where the trial was conducted. The authors meticulously extracted data from relevant studies and independently adjudicated discrepancies to ensure accuracy and reliability.

### 2.4 Outcomes measured

The reported stroke was the primary endpoint. The secondary endpoints included stroke reported by subtype (stroke etiological classification), either as ischemic (large-artery atherosclerosis, small-vessel occlusion, cardioembolic, stroke of other determined etiology, or stroke of undetermined etiology), haemorrhagic [intracerebral haemorrhage (ICH) or sub-arachnoid haemorrhage (SAH)], or minor stroke [transient ischemic attacks (TIAs)], and the diagnostic interventional technique applied for stroke management.

### 2.5 Quality assessment and risk of bias

To evaluate the methodological quality and potential bias in the included studies, each interventional trial was evaluated for risk of bias, and was assessed according to the criteria described in the Cochrane Quality Assessment Tool (13), which include selection, detection, performance, attrition, reporting, and others. Each domain was judged to be of either ‘low’, ‘high’, or ‘unclear’ risk, with the last category indicating uncertainty. Any study with a high score on the first three bias domains, which would indicate a ‘high risk’ trial, was considered to have low-quality evidence.

Each non-interventional trial was also evaluated for risk of bias, which was assessed according to the Risk of Bias In Non-randomized Studies of Interventions (ROBINS-I) Tool (14) with seven domains of bias, consisting of confounding, selection of participants, classifications of interventions, deviation from intended interventions, missing data, measurement of outcomes, and selection of reported results. Each domain was judged to be either ‘low risk’, ‘moderate risk’, ‘serious risk’, ‘critical risk’, or ‘no information’, with the last category indicating uncertainty in one or more key domains of bias. If a trial was judged to be at ‘critical risk’ of bias in at least one domain, it was considered to have low-quality evidence.

### 2.6 Statistical analyses

To summarize the characteristics of the included studies, descriptive statistics and frequency analysis were employed, presenting data in absolute numbers (*n.*) and percentages (%). These analyses were conducted using the Statistical Package for Social Sciences version 25, developed by IBM Corporation in Armonk, New York, United States (15). A narrative synthesis was utilized to amalgamate data from multiple studies, providing a comprehensive summary of each study’s data in both textual and tabular formats. To ensure consistency between different reviewers’ assessments, inter-rater reliability measures were implemented. Additionally, the risk of bias and quality analysis for interventional and non-interventional trials were conducted using Review Manager version 5.4.1 (16) and ROBINS-I (17) software, respectively, to generate graphical outputs.

## 3 Results

**FIGURE 1:**
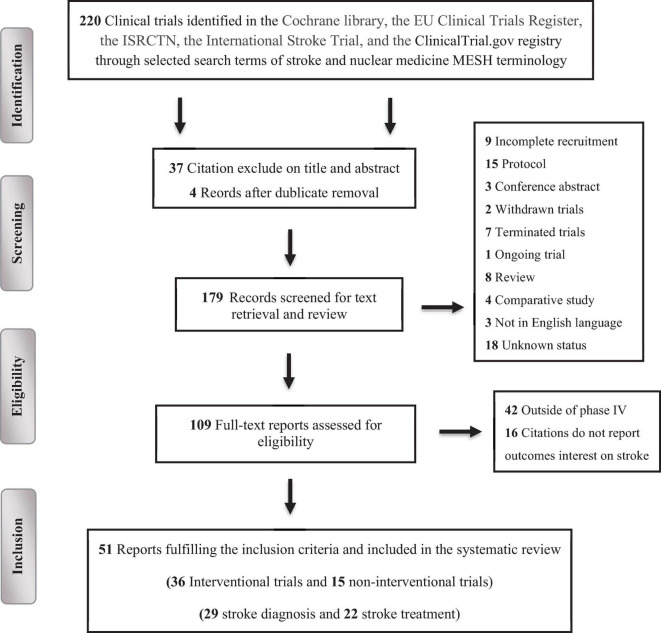
PRISMA flow chart depicting the selection process of the clinical trials included in the systematic review.

The systematic screening and filtration process initially identified 220 trials, comprising 178 (80.9%) interventional and 42 (19.1%) observational studies. Following the application of exclusion criteria, 111 (50.5%) trials were eliminated. The reasons for exclusion included: exclusion based on title and abstract (37; 16.7%), duplication (4; 1.7%), incomplete recruitment (9; 4.1%), protocol status (15; 6.7%), being conference abstracts (3; 1.2%), reviews (8; 3.5%), comparative studies (4; 1.7%), withdrawal (2; 0.8%), termination (7; 3.2%), ongoing status (1; 0.4%), unknown status (18; 8.2%), and non-English language (3; 1.3%). Out of the remaining 109 (49.7%) trials assessed for eligibility, 16 (7.5%) were excluded for not reporting stroke-related outcomes or being outside of phase IV (42; 19.3%). Ultimately, 51 (23.5%) clinical trials were deemed eligible and included in the final review, Figure 1.

### 3.1 Clinical trials characteristics

Out of the 51 included trials, 36 (70.6%) adopted an interventional clinical approach, with the remaining 15 (29.4%) trials adopting an observational clinical design. Of the observational trials, eight (53.3%) were cohort based and seven (46.7%) were case controlled (three (20%) were retrospective and 12 (80%) were time prospective), thereby shedding light on the clinical long-term impacts of nuclear medicine in association with the stroke population. For the interventional trials, two were phase I (5.6%), three were phase II (8.3%), one was phase III (2.8%), two were phase IV (5.6%), and 28 (77.7%) were completed, with the latter offering a more thorough understanding of the clinical applications of nuclear medicine for stroke diagnosis and management.

In terms of geographical distribution, one (1.9%) was enrolled in Egypt, 14 (27.5%) were registered in Korea and China, 14 (27.5%) were conducted in the United States countries, and 22 (43.1%) were carried out in European countries. For nuclear diagnostic trials, the median follow-up duration ranged from eight hours to 18 months; for nuclear management trials, the duration ranged from 12 hours to three months.

Detailed summary of the clinical trials design and baseline characteristics were compiled and presented (Tables 1, 2 for interventional; Tables 3, 4 for non-interventional) trials.

**TABLE 1:** Interventional studies for nuclear medicine applications in ischemic and haemorrhagic stroke diagnosis.

NCT Number	Study Title	Conditions	Interventions	Primary & secondary outcome measures	Enrollment	Study Design	Location
NCT 03943966 (18)	*In vivo* thrombus image with 18F-glycoprotein (GP)1, a novel platelet positron emission tomography (PET) radiotracer	Stroke, transient ischemic attack (TIA), pulmonary embolism, deep venous thrombosis, atherothrombosis, prosthetic valve thrombosis, myocardial infarction	Novel platelet radiotracer (18F-sodium fluoride) PET computed tomography (CT) scan	• The ratio of 18F-GP1 standardized uptake values in thrombus, as a marker of necrotic inflammation in human atherosclerosis, compared with the recorded in blood pool • To express the distribution of glycoprotein IIb/IIIa receptor within the cardiovascular thrombus in arterial and venous circulation within 6 months	73 adults including older adults	Randomized, parallel assignment	United Kingdom
ISRCTN 15483452 Brown et al. (19)	MINocyclinE to reduce inflammation and blood-brain barrier leakage in small vessel disease: a phase II, randomized, double-blind, placebo-controlled trial	Cerebral small vessel disease stroke	Minocycline antibiotic with anti-inflammatory properties versus placebo PET functional magnetic resonance imaging (fMRI) scan	• Reduction in inflammation measured by changes in cerebrospinal fluid biomarkers of inflammation • PET imaging to assess changes in regional cerebral blood flow and glucose metabolism, changes in clinical symptoms and disability, measured by scales such as the modified ranking scale or National institute of stroke (NIH) scale, assessed changes in brain function/structure using advanced imaging techniques like diffusion tensor imaging and functional MRI	44 Adults	Randomized, parallel, double masking	United Kingdom
NCT 03662750 (20)	Brain immune cell markers (TSPO) PET as a measure of post-stroke brain inflammation: a natural history imaging cohort	Acute ischemic stroke of moderate severity, inflammatory response	PET scan with radiotracers attach to brain immune cell markers (TSPO) and light up inflamed areas in brain versus MRI with gadolinium contrast	• PET-derived measures of TSPO radiotracer to track the extent and location of inflammation in the brain after stroke and peri-infarct areas (thalamus, hippocampi, amygdalae, midbrain) at day 90 (compared with day 15) • To explore whether the most inflamed areas in brain undergo the most post-stroke damage correspond to neurological and cognitive deficits (via measure blood inflammatory markers and genetic test for TSPO marker)	25 adults including older adults	Prospective cohort study, single masking, open-label	United Kingdom
VC21DIDS0085 Yoon et al. (21)	The electric simulation and proper positioning for mribased transcranial direct current stimulation in stroke	Hemiplegic chronic stroke	PET, functional MRI, and diffusion tensor imaging with optimized electrode placement and electrical parameters versus (placebo) or standard rehabilitation therapy	• Improvement in motor function of the affected limb, measured by standardized motor function scales such as the Fugl-Meyer Assessment or the Motor Assessment Scale • PET imaging to quantify changes in regional cerebral blood flow and glucose metabolism and functional MRI to map changes in brain activation patterns during motor tasks, and diffusion tensor imaging to evaluate changes in white matter integrity and neural connectivity	21 Adults	Randomized, parallel, double masking	Korea
NCT 03584425 (22)	Imaging laterality in chronic stroke patients	Chronic stroke with motor cortex lesion	Rasch modified version of fugl-meyer motor assessment, anatomical image acquisition, functional MRI task and acquisition	• Determine the neurobiological quantify a relationship between neural activity and motor performance compare glutamate/gamma-aminobutyric acid (GABA) ratio within contralesionally primary motor cortex using spectroscopy is higher in stroke patients than controls • Furthermore, in healthy controls prepulse inhibition will positively correlate with GABA/glutamate concentration	44 adults including older adults	Phase I, randomized, parallel masking	United States
Wu et al. (23)	Characterizing physiological heterogeneity of infarction risk in acute ischaemic stroke using MRI	Acute ischemic stroke	Diffusion-weighted and perfusion-weighted MRI were combined using a generalized linear model	• Assessment of physiological heterogeneity in infarction risk • Evaluation of blood flow and metabolic status on the variability of infarction risk within individual patients and improvement in personalized treatment strategies.	38 adults	Randomized, parallel, double masking	Denmark Germany
NCT 02452216 (24)	Using ferumoxytol-enhanced MRI to measure inflammation in patients with brain tumors or other conditions of the central nervous system	Ischemic and haemorrhagic stroke, brain injury, degenerative disorder, infectious disorder, vascular malformation, brain neoplasm	Ferumoxytol uptake with relaxation time (T2) MRI	• Measurements of iron concentration (indicative of ferumoxytol-enhanced) on T2 weighted MRI on day 1 • Determine number of inflammatory cells (macrophages) in resected biopsy sample at histopathology on days 2–4 for diagnostic, prognostic, and monitor of treatment response of current and future immunotherapies	10 Children, adults including older adults	Phase I, single masking, open-label	United States
Atchaneeyasakul et al. (25)	Intra-arterial ALD401 cell therapy is associated with reduction in stroke volume at 90 days in a subset of the RECOVER-stroke trial	Ischemic stroke	Intra-arterial administration of ALD401 cell therapy versus control group receiving standard care or a placebo	• The reduction in stroke volume, assessed at 90 days post-treatment. • Improve neurological functional recovery, recording any adverse events associated with intra-arterial administration of ALD401 cell therapy	11 adults	Phase III, randomized, parallel, double masking	United States
NCT 02809651(26)	Confounding factors in the detection of intracranial haemorrhage with the infrascanner	Ischemic stroke, intracranial hemorrhage, brain tumor, brain surgery after headache or head trauma	Infrascanner compared result of CT scan	• Utility determination of the positive and negative predictive value of the infrascanner for detecting of intracranial hemorrhage within 5 minutes • Delay of in hospital times, time from door to groin puncture (8 hours), rate of dramatic improvement, good functional outcome, and patients treated by endovascular treatment, modified ranking scale score	89 Children, adults including older adults	Randomized, parallel, triple masking	Belgium
Torres et al. (27) NCT 02405845	Prospective evaluation of carotid free-floating thrombus	Stroke, TIA	CT angiogram visual marker to distinguish unstable free-floating plaque from stable plaque	• Length of intraluminal filling defect on CT angiogram as a measure to distinguish between unstable free-floating thrombi from stable ulcerated plaque with 12 months follow up with clinician treatment strategies used to manage with antithrombotic treatment within 18 months	100 Children, adults including older adults	Single masking, open-label	Canada
Xu et al. (28)	Observation of the clinical effects of danhong injections combined with pitavastatin on blood lipid regulation in patients with ischemic strokes complicated with lipid abnormalities	Ischemic stroke complicated with lipid abnormalities	danhong injections combined with pitavastatin versus standard care using MRI and CT scans	• Clinical measurements of lipid levels, neurological function • MRI or CT scans to assess brain structure and ischemic damage • Carotid ultrasound to observe changes in carotid artery plaque and blood flow	116 Adults including older adults	Randomized, parallel, double masking	China
Featherstone et al. (29) ISRCTN 25337470	Carotid artery stenting compared with endarterectomy in patients with symptomatic carotid stenosis (International Carotid Stenting Study): a randomised controlled trial with cost-effectiveness analysis	Symptomatic atheromatous carotid artery stenosis	Carotid artery stenting versus carotid endarterectomy	• The incidence of any stroke or death within 30 days post-procedure • Incidence of disabling stroke, myocardial infarction, stroke or death in the initial 30-day period • Evaluated using MRI or CT scans to detect new or worsening ischemic lesions in the brain, indicating the occurrence of stroke or transient ischemic attacks	1713 Adults	Randomized, parallel, double masking	Worldwide
Skjetne et al. (30) NCT 02992821	Pocket sized carotid stenosis screening by junior doctors	Stroke, TIA, apoplexy	Bed-side pocket-size ultrasound (high frame rate tracking doppler) versus conventional pulsed wave doppler CT angiography for grades carotid stenosis	• Determine feasibility and reliability of bed-side ultrasound examinations accuracy of semi quantitative classification (non-significant atherosclerosis versus significant stenosis of carotid arteries) compared with reference imaging methods • Participants will be followed for a duration of hospital stay, an expected average of approximately within 4 days	75 adults including older adults	Single masking, open-label	Norway
NCT 00574457 (31)	Improving cardiovascular risk prediction use handheld carotid ultrasonography study	Atherosclerosis stroke	Carotid ultrasound	• To determine if non-sonographer health care professionals working in a community medical office practice setting can be trained to detect and evaluate subclinical atherosclerosis, reduce interventions and patient motivation to adhere to therapeutic recommendations within 2 years	355 adults including older adults	Phase IV, parallel masking	United States

**TABLE 2:** Interventional studies for nuclear medicine applications in ischemic and haemorrhagic stroke treatment.

NCT Number	Study Title	Conditions	Interventions	Primary and Secondary Outcome Measures	Enrolment	Study Design	Location
Haiting et al. (32)	Dengzhanxixin injection ameliorates cognitive impairment through a neuroprotective mechanism based on mitochondrial preservation in patients with acute ischemic stroke	Acute ischemic stroke with mild to moderate cognitive impairment	Dengzhanxixin injection versus placebo or standard care changes in brain mitochondrial function evaluated by PET scans	• Reduction in infarct volume measured by MRI, improvement in cognitive function assessed by standardized cognitive tests, survival rate of neuronal cells in the ischemic penumbra • Reduction in neurological deficits assessed by clinical scales NIH score, adverse effects related to dengzhanxixin injection administration	83 Adults	Randomized, parallel, double masking	China
Zhang et al. (33) WWH021	Effect of combined use of Buyang Huanwu decoction and olanzapine on clinical symptoms, neurological function, and degree of dementia in patients with vascular dementia after cerebral ischemic stroke	Vascular dementia post cerebral ischemic stroke	Buyang Huanwu decoction and olanzapine versus conventional group treated with olanzapine evaluated by PET scans	• Improvement in clinical symptoms measured by Mini-Mental State Examination and the Montreal Cognitive Assessment • Cerebral blood flow measured using single photon emission computed tomography (SPECT) and MRI perfusion to evaluate the effects of the treatment on cerebral perfusion, brain imaging changes assessed using MRI and CT scans to observe changes in brain structure and the extent of ischemic damage or brain atrophy	98 Adults	Randomized, parallel, double masking	China
Chen et al. (34)	The NeuroAiD II (MLC901) in vascular cognitive impairment study (NEURITES)	Vascular cognitive impairment post-stroke	NeuroAiD II (MLC901) versus placebo evaluated by PET and SPECT scans	• Improvement in clinical symptoms measured by Mini-Mental State Examination and the Montreal Cognitive Assessment • Using PET and SPECT scans to measure changes in cerebral perfusion and metabolic activity in the brain, and the use of MRI and CT scans to observe changes in brain structure, particularly in areas affected by vascular cognitive impairment	100 Adults	Phase II, randomized, parallel, double masking	China
NCT 04759950 (35)	Exercise the mind and brain. A multimodal intervention	Chronic stroke post-recovery	Behavioural multicomponent physical activity versus mindfulness stress reduction versus behavioural computerized cognitive train fMRI	• Clarify the neuroplasticity change (within 3 months after treatment at baseline) • The assessment includes medical, integrating cognitive, social, psychological evaluations, neuroimaging, and biological samples collection, stroke specific quality of life, time physical and activity questionnaire	141 adults including older adults	Randomized, parallel, double masking	Spain
Sui et al. (36)	Effects of trunk training using motor imagery on trunk control ability and balance function in patients with stroke	Post-stroke patients with motor dysfunction	Trunk training using motor imagery fMRI versus rehabilitation therapy	• fMRI to observe changes in brain activation patterns related to motor imagery tasks, indicating neural plasticity and adaptation in motor areas of the brain. • Diffusion tensor imaging (DTI): To evaluate changes in white matter integrity, potentially reflecting improvements in neural connectivity and structural reorganization in response to rehabilitation interventions.	100 Adults	Randomized, parallel, double masking	China
Ahmaed et al. (37)	Endovascular coiling versus surgical clipping in the treatment of ruptured anterior communicating artery aneurysm in Cairo university hospitals	Ruptured anterior communicating artery aneurysm with Hunt and Hess grade I, II or III	Endovascular coiling versus surgical clipping in spontaneous subarachnoid hemorrhage	• The effectiveness of aneurysm occlusion post-procedure, as assessed by follow-up imaging studies such as digital subtraction angiography and MRI • Using CT and MRI perfusion, and transcranial Doppler ultrasonography to assess changes in cerebral blood flow and perfusion parameters post-treatment	30 Adults	Prospective, randomized, parallel, double masking	Egypt
NCT 02791997 (38)	Audition after a lesion in migraine (audition postlesion)	Auditory process after brain (temporal or frontal) migraine	Neuropsychological tests versus neurophysiological markers (MRI, electro-, magnetoencephalography)	• To characterize auditory deficits after brain damage, investigate attention, short-term memory, sound-induced emotions • Percentages of correct responses, reaction times, and event-related potentials in neuropsychological tests up to two months	262 adults including older adults	Randomized, parallel masking	France
ISRCTN 80619088 (39)	MR CLEAN-NO IV treatment followed by IA treatment versus direct IA treatment for acute ischaemic stroke caused by a proximal intracranial occlusion	Acute ischaemic stroke with intracranial large vessel occlusion of anterior circulation	Intra-arterial treatment, or intra-venous thrombolysis with alteplase followed by intra-arterial treatment assessed via CTA or magnetic resonance angiography (MRA)	• Functional outcome measured by modified Rankin scale (at 90 days) • Reperfusion measured by extended thrombolysis in cerebral infarction (eTICI) score of 2b or more on final angiography of intra-arterial treatment, recanalization rate assessed with CT angiography (at 24 hours), and final infarct volume measured on cranial non-contrast CT (at 5-7 days) after randomisation	539 Adults	Randomized, parallel, double masking	Belgium, France, Netherlands
Walter et al. (40) NCT 00792220	Mobile stroke-unit for reduction of the response time in ischemic stroke	Ischemic stroke	Mobile stroke unit car with integrated CT scan versus occm	• Investigate whether a rescue car equipped with integrated CT contributes to a better stroke management by saving precious time until a therapeutic decision is made • Time between emergency call and therapy decision (CT, blood analysis, start of thrombolysis, time between symptoms, and functional status)	200 adults including older adults	Randomized, prospective, parallel masking	Germany
Koch et al. (41) NCT 01382862	Phantom-s: the pre-hospital acute neurological therapy and optimization of medical care in stroke patients	Acute ischemic and haemorrhagic stroke	Stroke emergency mobile unit (alarm-to-needle time) with and without availability of specially staffed ambulance with CT scan and point-of care diagnosis	• Implement a specialized stroke emergency mobile unit will reduce alarm-to-needle (initiation of thrombolysis), alarm-to-imaging, alarm-to-point-of-care laboratory diagnostics a minimum of 20 min, in case of distal carotid occlusion, proximal MCA, space occupying cerebellar haemorrhage (at least 6 ml), intraventricular, and symptomatic ICH (36 hs after symptom onset up to 3 months), in-hospital mortality, functional outcomes, costs effectiveness • Telephone follow-up after 3 months	614 adults including older adults	Phase II, prospective, randomized, parallel masking	Germany
Al-Shahi Salman et al. (42)	Effects of antiplatelet therapy on stroke risk by brain imaging features of intracerebral haemorrhage and cerebral small vessel diseases: subgroup analyses of the RESTART randomised, open-label trial	Intracranial haemorrhagic stroke (ICH)	Antiplatelet therapy on diffusion-weighted imaging (DWI) or diffusion tensor imaging (DTI) versus standard care	• The incidence of recurrent stroke or other vascular events. • Functional outcomes, mortality rates, and adverse events related to treatment.	537 Adults	Randomized, parallel masking, open-label	United Kingdom
NCT 03038087 (43)	A study to test the sense device in patients with intracranial haemorrhage	Haemorrhagic stroke	Feasibility of sense device for bleed monitoring versus CT scan	• To assess a positive predictive value of the SENSE device for detecting number of patients correctly identified with haemorrhage expansion (> 3 ml increase in volume), serious device-related adverse events, or seizure (within 12-72 hs, up to 6 weeks)	10 adults including older adults	Single masking, open-label	United States
NCT 03827720 (44)	Early feasibility study of the sense device	Ischemic and haemorrhagic stroke	Preliminary estimation of sense device safety in large vessels occlusion versus non	• Correlation of sense signal (within 24 hs of stroke symptom), change in received power measured and accuracy of sense algorithm, with diagnostic CT scan for stroke severity (location), haemorrhage volume, cerebral oedema, and acute ischemic stroke with large vessels occlusion monitors within 45 min	20 adults including older adults	Randomized, parallel masking	United States
NCT 02258789 (45)	Assessment and training visio-spatial neglect in a virtual reality environment	Hemispatial neglect in chronic stroke (right-sided ischemia)	Virtual reality technology versus method	• Stimulating attention network: top-down scan training in a 3d game, combined with intense visual, audio, and tactile bottom-up stimulation, motor for home rehabilitation and tele-medicine approaches • Neglect assessment of clinical and behavioural score changes, star cancellation test, line bisection, baking tray, Posner task, extinction, catherine Bergego scale, within 1 week after intervention • Catherine bergego scale, change in score in spatial attention daily life activities, self-report from patient and relative within 6 month follow up	15 adults including older adults	Single masking, open-label	Sweden
NCT 06341777 (46)	Multisensory telerehabilitation for visual field defects	Visual field defect follows stroke hemianopia, brain injuries	Behavioral: audio-visual training telerehabilitation	• Verifying the feasibility and efficacy of a novel telerehabilitation use a multisensory scan therapy • Measure its effects on visual functions, daily activities, looking for neural indicators of the therapy-induced improvements within 6 months follow up	72 Children, adults including older adults	Randomized, parallel, single masking	Italy
NCT 05466487 (47)	Non-invasive brain stimulation as innovative treatment for chronic neglect patients	Spatial neglect in chronic stroke to promote function recovery	Transcranial alternat current stimulation behavioral visual scan train versus neglect train with sham stimulation	• To exploit principles of non-invasive brain stimulation to promote functional recovery in chronic stroke suffered from neglect symptoms • Combine evidence-based visual scan trained with transcranial alternating current stimulation treatment at alpha frequency • Computerized visual detection, star cancellation, quality of search score, baking tray, neuropsychological test, neglect questionnaire (at baseline and after 1, 9, 18 weeks, 3 months after termination of training session)	22 adults including older adults	Randomized, double blind, parallel, quadruple masking	Netherland
NCT 03302741 (48)	3d image innervation zone distribution in spastic muscles from high-density surface electromyography (EMG)	Muscle spasticity after stroke, amyotrophic lateral sclerosis	New 3d image guide botulinum neurotoxin versus physical therapy	• Spasticity assessed by reflex torque of elbow flexors, modified Ashworth scale, receive a total of 60 degrees (50-100 sec at 1 day baseline to 3 weeks) of computer-controlled elbow extension stretching at different speeds	17 adults including older adults	Phase IV, randomized, parallel, masking	United States
Lee et al. (49) NCT 03048968	The effect of a walking assist robot on gait function and brain activity in stroke patients and elderly adults	Stroke	New wearable hip assist robot technology on gait, sit-to-stand movement, stair climbing and treadmill gait versus sit-to-stand movement and treadmill gait versus gait enhancing mechatronic system versus treadmill	• To investigate the effects of new wearable hip assist robot, gait enhancing and motivating system Cortical activation measured by functional near-infrared-spectroscopy during treadmill gait (30 min), berg balance (sitting and standing balance during transfers), fall efficacy, and modified ranking scale to assess static balance and fall risk, 10 items questionnaire	40 adults including older adults	Randomized, parallel masking	Korea
Lazaridou et al. (50)	Diffusion tensor and volumetric MRI using MR-compatible hand-induced robotic device suggests training-induced neuroplasticity in patients with chronic stroke	Chronic stroke	MR-compatible hand-induced robotic device evidenced by diffusion tensor imaging and volumetric MRI	• To investigate whether the use of an MR-compatible hand-induced robotic device in chronic stroke patients could lead to observable neuroplastic changes as evidenced by diffusion tensor imaging and volumetric MRI • To assess changes in white matter integrity and brain volume before and after the intervention • Any functional improvements in hand motor function or other relevant clinical outcomes after undergoing the robotic device training	19 Adults	Randomized, parallel masking	United States
NCT 05844722 (51)	Effectiveness of mindfulness cognitive therapy undergoing post-stroke rehabilitation	Stroke rehab mindfulness	Behavioral mindfulness cognitive therapy versus usual care	• Efficacy of mindfulness changes on beck severity of depression and anxiety state-trait inventory (at baseline, 6 weeks, 3 months) • Global index of attention and perception is calculated using a five-facet mindfulness questionnaire with 39 items scored to assess sustained attention, sensorimotor impairment, functional independence of basic activity of daily living	93 adults including older adults	Randomized, parallel masking	Hungary
Liu et al. (52)	Clinical study of opposing needling combined with eye acupuncture for treatment of muscle spasm post-stroke hemiplegia patients	Post-stroke hemiplegic patients	Acupuncture treatment, needling combined with eye acupuncture, versus basic rehabilitation treatment	• To assess the effectiveness of opposing needling combined with eye acupuncture therapy in reducing muscle spasms. • Improvements in functional mobility and activities of daily living, patient-reported measures of pain reduction, quality of life improvements, or satisfaction with the treatment regimen.	69 Adults	Randomized, parallel masking	China
NCT 03648957 (53)	Lifestyle counselling as secondary prevention in patients with minor stroke or TIA	Acute minor ischemic stroke, TIA, haemorrhagic stroke	Lifestyle counselling targeting smoking, physical activity, adherence to preventive medication as secondary prevention versus usual care	• Evaluate the feasibility and effect of an early initiated counselling intervention changes on resting systolic blood pressure and adherence rate (from baseline and 3 months follow up) • Explored barriers and facilitators for health behavior after stroke, include perceived needs and social support	40 adults including older adults	Randomized, parallel masking	Denmark

**TABLE 3:** Observational studies for nuclear medicine applications in ischemic stroke diagnosis.

NCT Number	Study Title	Conditions	Interventions	Primary/Secondary Outcome Measures	Enrollment/ Age	Study Design	Location
NCT 00001324 (54)	PET scan to study brain control of human movement	Ataxia, cerebrovascular accident, movement disorder, tremor	PET scan	• Investigate brain activity and regional cerebral blood flow using radioactive water (h215o) and PET scan • Stimulate the areas of brain responsible for voluntary motor activity and sensations	510 Children, adults	Time prospective	United States
Boukrina et al. (55) NCT 03349411	Spatial neglect and delirium after stroke	Stroke, delirium, spatial neglect (right brain ischemia)	Diffusion, structural MRI, Florida mental status exam, Montreal cognitive assess, Kessler foundation neglect	• Estimate the correlation of right-dominant brain networks as brain biomarkers of behavioural signs and severity of delirium detection from spatial neglect such as arousal, attention, activity, structural integrity, functional connectivity, confusion assessment method within 1 week	45 adults, older adults	Patient registry, cohort, time prospective	United States
NCT 03974464 (56)	Copeptin and the S-100b protein in stroke	Stroke with vertigo in emergency department	S-100 b protein assay, copeptin, and brain MRI	• Evaluated the value of using copetin and protein S-100b to eliminate stroke diagnosis in patients presenting with vertigo in emergency departments	151 adults, older adults	Time prospective	United States
Lyu et al. (57) NCT 02580097	MR-based stroke mechanism and future risk score	Acute stroke	Diffusion-weighted image MRI	• Predicting multimodal for imaging evaluation of hemodynamic status and antegrade flow of final infarct volume and growth within 7 days to 1 month • Follow up by assessment of modified Rankin score within 3 months and neurological mortality outcome assessment within 1 year	961 adults, older adults	Patient registry, Case-only, time prospective	China
NCT 02576743 (58)	Pushing spatiotemporal limits for 4d flow MRI and dynamic MRA in brain at ultra-high field	Stroke aneurysms and arteriovenous malformations	4d flow MRI data acquired at ultra-high field strength (7 tesla)	• Investigate the brain blood flow hemodynamic with high spatiotemporal resolution, peak blood velocity, and wall shear stress parameters within 2 years	90 adults, older adults	Case-control, time prospective	United States
NCT 02033291 (59)	Blood-brain barrier quantification in cerebral small vessel disease	Cortical stroke, cerebral small vessel disease, Alzheimer’s disease, vascular dementia, hypertension, diabetes	Dynamic contrast-enhanced brain MRI	• Pharmacokinetic parameter values evaluated as a function of scan duration to obtain the shortest scan duration without compromising the reliability of blood brain barrier permeability • Quantify outcome measures to determine the reproducibility of dynamic contrast enhanced MRI method (within day 1 and 2, and 4 weeks)	17 adults, older adults	Case-only, time prospective	Netherland
Dale et al. (60) NCT 02308605	Smartcap stroke study: a field deployable blood test for stroke	Stroke	Smartcap for purines measure as early biomarker of brain ischemia	• To developed new biosensor technology to rapidly measure purines (cellular starved of oxygen and glucose) in freshly drawn blood • Identify ischemic versus haemorrhagic strokes by combining purine measurements, in blood within 30 minutes down to 5 minutes, CT scans within 24 hours to 7 days	217 Children, Adults, older adults	Case-control, time prospective	United Kingdom
NCT 04983927 (61)	Evaluation of clinical decision support system for scoring of Alberta stroke program early CT Score (ASPECT) score using CT image	Ischemic stroke	Caspects, large vessel occlusion who undergo endovascular revascularization	• Evaluate efficacy of caspects software for scoring ASPECT compared to the sensitivity and specificity by automatically analysing brain CT and mean difference calculated by human experts and caspects (within 4 weeks) for clinical decision support system	326 adults, older adults	Case-control, time prospective	Korea
NCT 01764763 (62)	Intraoperative epiaortic scan for preventing early stroke after off-pump coronary artery bypass	Coronary artery occlusive disease	Epiaortic ultrasound as an intraoperative tool to assess ascending aorta disease early stroke after off-pump coronary artery bypass	• Predicting number of incidences of early stroke from surgery to discharge with an expected average of 5 weeks	2292 adults, older adults	Cohort, time retrospective	Korea
NCT 02793362 (63)	Measurement of sarcopenia post-stroke rehabilitation outcome	Stroke sarcopenia	Effect of sarcopenia on post-stroke hemiplegia recovery	• Quantify post-stroke loss of muscle mass validity using echo intensity elastography • Change in Korean modified Barthel index score, berg balance, fatigue, functional oral intake, motricity index scales, from initial evaluation to the final evaluation within 8, 12, and 16 weeks	70 adults, older adults	Cohort, time prospective	Korea
Langer et al. (64) NCT 05145855	The effects of offline anosognosia for spatial neglect on neglect rehabilitation	Hemispatial neglect, hemiplegia, right hemispheric stroke, anosognosia	Patient-tailored multimodal hemispatial neglect rehabilitation	• Investigate relationship between decrease in anosognosia for neglect, improvement of spatial deficits using catherine bergego scale activities of daily living • Prevalence of extinction on visual, auditory, tactile double simultaneous stimulation test, functional ambulation, independence measurement, Brunnstrom stage of upper and lower extremity sensorimotor functions, recovery status before and after rehabilitation	85 adults, older adults	Cohort, time retrospective	Turkey

**TABLE 4:** Observational studies for nuclear medicine applications in haemorrhagic stroke diagnosis.

NCT Number	Study Title	Conditions	Interventions	Primary/Secondary Outcome Measures	Enrollment/ Age	Study Design	Location
NCT 01829386 (65)	Non-heme iron load quantification in the brain MRI of patients with stroke	Haemorrhagic stroke	MRI scan with non-heme iron levels	• Create a non-invasive imaging modality of iron levels assessments for brain damage estimation • Validated quantification within 30 days	34 adults including older adults	Cohort, time prospective	United States
NCT 02033291 (59)	Blood-brain barrier quantification in cerebral small vessel disease	Cortical stroke with cerebral hamorrhage	Dynamic contrast enhanced brain MRI	• Pharmacokinetic parameter values evaluated as a function of scan duration to obtain the shortest scan duration without compromising the reliability of blood brain barrier permeability • Quantification ki and vb outcome measures to determine the reproducibility of dynamic contrast enhanced MRI method (within day 1 and 2, and 4 weeks)	17 adults including older adults	Case-only, time prospective	Netherland
NCT 06213870 (66)	Screening of endovascular thrombectomy with MRI in acute ischemic stroke	Symptomatic intracranial hamorrhage	Fluid-attenuated inversion recovery sequence vascular hyperintensity-diffusion-weighted MRI image mismatch preoperative and post-endovascular thrombectomy	• Predict the consistency between MRI and perfusion examination in determining endovascular thrombectomy indications and functional independence rate, National institutes of health stroke scale (NIHSS) > 4 scores, modified Rankin scale 0–2 scores at 90 days	309 adults including older adults	Cohort, time retrospective	China
Chibbaro et al. (67) NCT 01113645	Impact of cranioplasty on cerebral perfusion	Decompressive craniectomy post head injuries, subarachnoid hamorrhage, intra-cerebral hamorrhage, cerebral thrombosis, infarction, malignant middle cerebral artery	Cerebral CT perfusion and trans-cranial doppler evaluations	• Evaluate the impacts of local and global cerebral hemodynamic and blood flow outcomes as prognostic factors, by Glasgow outcome score, frontal assessment battery, and mini mental state examination scores, within 1 week prior and 6 and 24 weeks post-cranioplasty	20 adults including older adults	Cohort, time prospective	France
NCT 04554368 (68)	A novel score to predict risk of symptomatic intracerebral hamorrhage	Intracerebral hamorrhage post-stroke thrombectomy	Intra-arterial contrast (Henan-PRIHS) enhanced flat detector CT	• Predicting the stratification risk of symptomatic intracranial hamorrhage scores in affected hemisphere within 5 days	300 adults including older adults	Patient registry, case-only, time prospective	China

### 3.2 Trials population

The majority of the included trials were conducted with stroke participants ranging from ≥ 18 and 65 years of age, and only six (16.7%) trials included pediatric patients ≤ 17 years old. The majority of participants were male (6,836; 60.7%). In total, these trials included 11,262 participants who were exclusively diagnosed with either ischemic (9,232; 82.0%) or haemorrhagic (2,030; 18.0%) strokes.

### 3.3 Risk of bias assessment

**FIGURE 2:**
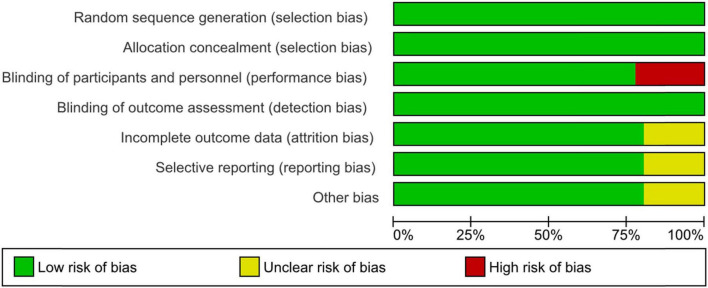
Risk of bias graph: review authors’ judgments about each risk of bias item presented as percentages across all included interventional trials in the systematic review.

Among the 36 interventional trials, 29 (80.6%) were randomly allocated with a blinded treatment allocation strategy and scored with a low risk of selection bias. However, seven (19.4%) trials employed an open-label design and scored with a high risk of performance bias. In terms of masking, the primary intervention model used was double blind parallel assignment (27; 75.0%), followed by single (seven; 19.4%), triple (one; 2.8%), and quadruple (one; 2.8%) masking techniques. The risk of attrition and reporting biases was unclear in seven single masking trials (19.4%), Figure 2.

**FIGURE 3:**
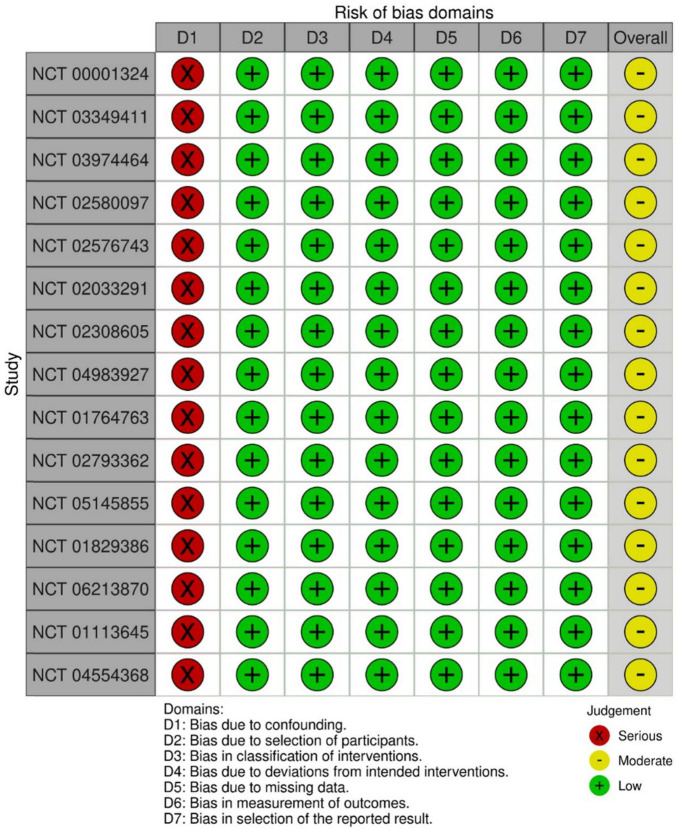
Risk of bias graph: review authors’ judgments about each risk of bias item presented as percentages across all included non-interventional trials in the systematic review.

For the observational trials, 15 (41.7%) were measured by confounding variables and scored with a serious risk of bias. Generally, the trials were pro- or retrospectively recruited with a consecutive series of participant selections. They did not deviate from the intended intervention of interest, were well defined, and were based solely on what was collected at the time of intervention. In terms of missing outcomes, participants were selected and chosen based on their outcome data. It is unlikely that the outcome measures were influenced by knowledge of the intervention on the part of the trial’s participants; methods of assessment were comparable across groups, and any error in outcome measurement was only minimally related to intervention status. Selection of the reported results was based on clinical knowledge that appeared not to be based on the intervention, outcome, or multiple analyses. Therefore, the overall risk of bias was moderate, without adjustment for confounding (Figure 3).

### 3.4 Outcome measures

All 51 appraised trials were specifically designed to assess strokes as primary and secondary outcomes, and there was an adequate description of the outcome data. Specifically, 14 (38.9%) interventional trials examined the impacts of nuclear medicine on ischemic stroke diagnosis (of which two examined haemorrhagic stroke as well), whereas an additional 22 (61.1%) examined the impacts on ischemic stroke treatment (of which six also examined haemorrhagic stroke). Six trials focused on acute stroke conditions, yet the predominant focus was on chronic stroke, examined in 30 trials. This substantial emphasis on chronic stroke encompassed 83.3% of patients with large-artery atherosclerosis and cardioembolic conditions. The remaining 16.7% comprised patients with cSVD (one trial) and TIAs (four trials). For the observational trials, 11 (73.3%) examined the impacts of nuclear medicine on ischemic stroke diagnosis (of which one examined haemorrhagic stroke), and an additional four trials (26.7%) examined the impacts on haemorrhagic stroke diagnosis. The most frequently examined stroke condition was acute hemorrhage, accounting for a significant group with symptomatic ICH (five; 33.3%), but a minority with SAH (one; 6.7%).

Regarding the diagnostic interventional techniques, the trials investigating the impact of nuclear medicine on stroke diagnosis employed various approaches: four utilized PET CT/MRI radiotracer scans, four employed diffusion- and perfusion-weighted MRI, three used CT angiography, one used Infrascanner, and two utilized carotid angiography ultrasounds. For stroke treatment, three trials utilized PET and SPECT scans, five employed functional MRI angiography, five used CT angiography, one implemented a novel 3D image guide, two used sensory devices, three incorporated audiovisual reality technology scans, two utilized robotic technology, and one trial explored the use of acupuncture. However, the observational trials focused solely on the impacts of stroke diagnosis, employing various techniques: one PET radiotracer scan, seven MRIs, three CTs, one epiaortic ultrasound, one new Smartcap device, and two multimodal rehabilitation approaches. The trials compared these diagnostic interventions against other methods used in stroke management.

### 3.5 Outcome results

Trial outcome results are detailed in Tables 1, 2 for interventional and Tables 3, 4 for non-interventional trials. The description of the stroke outcome data is organized here according to their applications in clinical practice: (1) diagnosis, (2) treatment decisions, (3) predictive biomarkers, outcomes, and prognosis, (4) secondary prevention, and (5) rehabilitation.

#### 3.5.1 Nuclear medicine clinical applications for ischemic stroke diagnosis

##### 3.5.1.1 Implementation of pre-hospital acute stroke care notification and therapy protocol

The implementation of a mobile stroke unit (MSU) with an integrated CT scan led to a significant reduction in the time from symptom onset to the initiation of appropriate treatment, such as thrombolysis or EVT, within 35 min (40). This reduction in response time was associated with improved clinical outcomes, including reduced disability and mortality rates among ischemic stroke patients. Although MSUs facilitate on-site stroke diagnosis and the initiation of time-critical interventions, they also contribute to more efficient and streamlined stroke care delivery. Furthermore, the implementation of pre-hospital acute neurological therapy protocols with integrated CT scans led to significant improvements in various aspects of stroke care, with mean time differences in alarm-to-treatment time of 34.7 minutes compared with conventional care (41). These improvements included reduced time to treatment initiation, increased rates of thrombolysis administration, and enhanced adherence to established stroke treatment guidelines. In addition, pre-hospital acute neurological therapy interventions were associated with improved clinical outcomes, including reduced disability and mortality rates among stroke patients. Together, these findings underscore the importance of innovative approaches, such as MSUs, in optimizing acute stroke care, minimizing treatment delays, as well as highlighting the potential benefits of implementing structured protocols for pre-hospital stroke management.

##### 3.5.1.2 Distinction between stroke mimics, healthy controls, and acute stroke

An MRI-based method for identifying and quantifying non-heme iron levels deposited in brain tissue, particularly in areas associated with ischemic injury and neurodegeneration, following haemorrhagic stroke, has been validated (65). The degree of non-heme iron accumulation was found to be correlated with the severity of stroke-related neurological deficits and functional impairment. These findings suggest that non-heme iron accumulation may contribute to the pathophysiology of stroke and its associated complications, potentially serving as a biomarker for assessing disease severity and prognosis in stroke patients. The quantitative assessment of non-heme iron load using MRI techniques holds promise for elucidating the underlying mechanisms of stroke-related brain damage and may inform the development of novel therapeutic interventions targeting iron dysregulation in stroke.

##### 3.5.1.3 Distinction between intracerebral hemorrhage and ischemic stroke

Potential confounding factors influencing the accurate detection of ICH can be identified using the Infrascanner. Several factors significantly impact the reliability of ICH detection, including patient age, Glasgow Coma Scale (GCS) score, and the presence of scalp hematoma (26). Specifically, older age and lower GCS scores were associated with decreased sensitivity of the Infrascanner in detecting ICH, while the presence of scalp hematoma posed challenges in distinguishing between superficial bleeding and ICH. A promising outcome was that the Smartcap blood test showed high accuracy in diagnosing stroke. The blood test utilized a rapid and portable device capable of detecting specific biomarkers and measuring purine levels in freshly drawn blood associated with stroke, enabling swift diagnosis and initiation of appropriate medical interventions in pre-hospital and emergency care settings (60). Specifically, the Smartcap blood test showed potential for distinguishing between ischemic and haemorrhagic strokes after 24 hours. Overall, these findings emphasize the importance of considering various clinical factors when interpreting Infrascanner results to improve their diagnostic accuracy and reliability in emergency settings for timely and appropriate patient management. They also suggest that the Smartcap blood test holds promise as a valuable tool for improving stroke diagnosis and triage, ultimately leading to better patient outcomes and a reduced disability burden associated with stroke.

##### 3.5.1.4 Identify the etiology of stroke

Incorporating MRI-based stroke mechanisms such as large artery atherosclerosis, cerebral small vessel disease (cSVD), and cardioembolism into a comprehensive risk score significantly enhanced the accuracy of predicting future stroke events (57). This advanced MRI-based risk score, powered by machine learning, identified individuals at high risk of recurrent stroke with a collateral-core ratio, achieving a mean net reclassification index of 52.7% ± 32.7%, thereby facilitating targeted preventive interventions and personalized management strategies. Furthermore, ultra-high-field MRI significantly improved the spatiotemporal resolution of 4D flow nuclear imaging and dynamic magnetic resonance angiography (MRA), allowing for more detailed visualization of cerebral blood flow dynamics and vascular anatomy (58). This higher resolution enabled the detection of smaller vessels and subtle flow abnormalities, enhancing diagnostic capabilities for evaluating cerebrovascular pathologies. In another vein, the novel 18F glycoprotein (GP) 1 PET radiotracer, which binds with high affinity to the GP IIb/IIIa receptor involved in platelet aggregation, demonstrated effective *in vivo* thrombus detection across various preclinical models (18), offering superior resolution compared to traditional MRI and CT modalities. Lastly, minocycline’s efficacy, antibiotic with known anti-inflammatory properties, in reducing inflammation was assessed using PET (26) and MRI dynamic contrast-enhanced brain imaging (59), revealing significant blood-brain barrier (BBB) permeability alterations in patients with cSVD compared to healthy controls, indicating increased leakage of contrast agents into the brain parenchyma.

These findings underscore the potential of MRI-based risk stratification tools to optimize secondary prevention efforts and reduce recurrent strokes, the promise of ultra-high-field MRI for advancing cerebrovascular diagnostics, the utility of 18F-GP1 PET imaging for non-invasive thrombus detection, and the critical role of BBB dysfunction in the pathophysiology of cSVD, contributing to the progression of white matter hyperintensities, lacunar infarcts, and other neurovascular abnormalities.

#### 3.5.2 Nuclear medicine clinical applications for ischemic stroke treatment

A novel scoring system was created for an enhanced flat detector CT, which effectively stratified patients based on their risk of developing symptomatic ICH following reperfusion therapy (68). The scoring system incorporated various clinical and imaging factors, such as age, stroke severity, baseline neuroimaging findings, and comorbidities, to accurately estimate individualized risk profiles for symptomatic ICH. It showed good discrimination and calibration in both the derivation and validation cohorts, indicating its reliability and generalizability for clinical use. This system may serve as a valuable tool for clinicians in risk-stratifying patients undergoing reperfusion therapy for acute ischemic stroke, facilitating informed decision making, and optimizing patient outcomes by mitigating the risk of symptomatic ICH.

##### 3.5.2.1 Estimate the time onset of stroke and the volume of ischemic penumbra and the core

The integration of a clinical decision support system into stroke care has been shown to enhance the accuracy and consistency of interpreting the Alberta Stroke Program Early CT Score on CT images (61), leading to improved interobserver agreement and reduced variability among clinicians. Additionally, advanced MRI techniques like perfusion-weighted imaging and diffusion-weighted imaging (DWI) MRI (30) can identify varying levels at high risk of infarction in stroke patients by revealing significant within-patient heterogeneity influenced by factors such as blood flow and metabolic status, thus facilitating detailed, patient-specific assessments that improve personalized treatment strategies for acute ischemic stroke.

##### 3.5.2.2 Predict recanalization following intravenous thrombolysis

Vascular hyperintensity-diffusion-weighted MRI (66) or CT angiography (54) can be used as a screening tool to identify eligible candidates for EVT among patients with acute ischemic stroke (39) by accurately detecting large vessel occlusions and assessing the extent of ischemic brain injury. Overall, incorporating MRI or CT angiography screening into the acute stroke workflow may enhance the efficiency and accuracy of patient selection for EVT.

##### 3.5.2.3 Predict haemorrhagic transformation in ischemic stroke

The effect of antiplatelet therapy on stroke risk identified by brain imaging features like ICH and cSVD employs DWI or diffusion tensor imaging (DTI) (42) to monitor alterations in white matter integrity and structural damage progression. These results underscore the significance of personalized treatment strategies aligned with brain imaging characteristics to enhance stroke risk management effectively.

The sense device for bleed monitoring effectively allowed for the early detection of seizures activity, cerebral edema, and monitoring hemorrhage expansion in patients with acute ICH (43). The continuous monitoring provided by the sense device allowed for the early detection of changes in brain activity within six hours of symptom onset, providing findings similar to CT scans, facilitating prompt medical attention, and potentially improving stroke patient outcomes (44). These findings suggest that the sense device holds promise as an innovative solution, with high sensitivity and specificity, for monitoring patients with ICH and preventing seizure-related complications.

#### 3.5.3 Nuclear medicine clinical applications for ischemic stroke prognosis

The dynamic changes in PET scans with radiotracers of TSPO, which are putative markers of microglia activation and neuroinflammation in stress-related brain inflammation, can indicate a fluctuation in the inflammatory response following stroke over a period of 90 days (20). Specifically, TSPO PET imaging showed elevated binding in the acute phase of stroke distant from the infarct area (e.g., thalamus, hippocampi, amygdalae, and midbrain), which gradually decreased over time, suggesting resolution of inflammation when compared with day 15 (mean SD g/mL 0.89; *P* = 0.04). However, a subset of patients exhibited persistent or even increased TSPO binding, indicating ongoing or exacerbated inflammation that corresponded to the cognitive and neurological deficits experienced by stroke patients and thus poorer functional outcomes. These findings highlight the potential of TSPO PET imaging as a valuable tool for assessing post-stroke inflammation dynamics and its implications for stroke treatment strategies and patient prognosis.

Additionally, the impact of intra-arterial ALD401, a stem cell therapy product derived from autologous bone marrow mononuclear cells, on stroke volume reduction has had a significant reduction in stroke volume at 90 days post-treatment 22.9 ± 15.5 ml (*P* = 0.046) in ischemic stroke patients (25). These results suggest that ALD401 cell therapy may offer a beneficial intervention for reducing stroke-related brain damage in certain stroke patients.

##### 3.5.3.1 Predict the early peri- and post-operative stroke complications

Endovascular coiling demonstrated a lower complication rate and shorter recovery time compared to surgical clipping, though both techniques were effective in achieving aneurysm occlusion in ruptured anterior communicating artery aneurysms, suggesting the minimally invasive nature of coiling offers superior patient outcomes (37). Similarly, the use of intraoperative epiaortic scanning significantly reduced early stroke complications in off-pump coronary artery bypass surgery by allowing real-time visualization of aortic atherosclerosis and embolic sources, thus enhancing perioperative safety and outcomes (62). Additionally, cranioplasty performed within 6 to 28 hours post-head injury markedly improved cerebral perfusion parameters, such as cerebral blood flow and cerebrovascular reactivity, with younger patients and those treated earlier showing near-normal perfusion levels, as evidenced by cerebral CT perfusion and transcranial Doppler evaluations (67). These findings underscore the critical role of advanced intraoperative imaging techniques and timely surgical interventions in optimizing patient recovery and improving neurological outcomes in various cerebrovascular and cardiovascular conditions.

#### 3.5.4 Risk stratification for secondary prevention

##### 3.5.4.1 Transient ischemic attacks

A lifestyle counseling intervention was found to significantly reduce modifiable risk factors for stroke recurrence, such as hypertension, diabetes, smoking, and physical inactivity, among patients with minor stroke and TIA (42). Participants who received counseling demonstrated greater adherence to healthy lifestyle behaviors, including regular physical activity, balanced diet, and medication compliance, which contributed to a reduced risk of recurrent stroke and improved overall prognosis. These findings underscore the importance of lifestyle modifications as key components of secondary prevention strategies for mitigating stroke risk and optimizing long-term outcomes in individuals with minor stroke and TIA.

##### 3.5.4.2 Large artery atherosclerotic stroke

Pocket-sized ultrasound scanners have demonstrated high feasibility and reliability for assessing carotid artery intima-media thickness and the presence of plaques, showing sensitivity of 97%, specificity of 63%, and positive and negative predictive values of 93% and 87%, respectively, even when used by inexperienced operators (30). This suggests that such portable devices could be valuable tools for junior doctors, enabling early detection of carotid artery stenosis and subclinical atherosclerosis, thereby potentially reducing the need for more advanced and costly imaging procedures (31). Integrating these devices into routine clinical practice, especially in settings with limited access to specialized equipment, could enhance the efficiency and accessibility of carotid artery screening for cardiovascular risk prediction.

Meanwhile, in a study comparing carotid artery stenting and endarterectomy in patients with symptomatic carotid stenosis, both procedures were effective in stroke prevention, though carotid stenting was associated with a higher incidence of procedural complications (29). The combined use of MRI and CT angiography and portable ultrasound devices may provide comprehensive strategies for optimizing carotid artery disease management to assess restenosis rates and cerebral ischemic lesions, contributing to a cost-effectiveness analysis of the two treatments.

##### 3.5.4.3 Embolic stroke

Carotid free-floating thrombus (FFT) was primarily detected in stroke patients with atherosclerosis and significant carotid stenosis. Symptomatic carotid FFT patients face a higher risk of embolic events than those with asymptomatic thrombi. CT angiogram identified morphological characteristics, such as thrombus size and mobility, as significant embolic risk predictors. A cranial-caudal length threshold of 3.8 mm distinguished FFT from plaque with 88% sensitivity and 83% specificity (27). Additionally, the combination of danhong injections and pitavastatin significantly improved lipid profiles and reduced ischemic lesions in ischemic stroke patients, as evidenced by MRI and CT angiogram scans. Carotid ultrasound showed decreased artery plaque and enhanced cerebral blood flow, suggesting improved recovery and reduced stroke risk in patients receiving this combined therapy (28). Early detection and risk stratification are crucial for guiding management strategies and preventing embolic complications.

##### 3.5.4.4 Predict the stroke severity and outcomes

The levels of copeptin and the calcium-binding protein B (S-100B protein), which function as biomarkers for assessing stroke severity and predicting clinical outcomes, were significantly elevated in stroke patients compared to healthy controls (56). Higher levels of copeptin and S-100B on MRI were associated with increased stroke severity, as evidenced by higher National Institutes of Health Stroke Scale (NIHSS) scores, and were predictive of poorer clinical outcomes, including an increased risk of mortality and disability following a stroke. Furthermore, ferumoxytol-enhanced MRI successfully detected and quantified inflammatory activity within the central nervous system (CNS), which provided valuable insights into disease progression and treatment response (24). Specifically, the imaging technique demonstrated enhanced contrast between inflamed and non-inflamed tissues. Ferumoxytol-enhanced MRI has shown potential as a non-invasive biomarker for monitoring therapeutic interventions and assessing treatment efficacy in patients with brain tumors and other CNS disorders, thereby facilitating more personalized and timely clinical management strategies.

#### 3.5.5 Predict clinical improvements post-stroke rehabilitation

##### 3.5.5.1 Predict the post-stroke cognitive recovery

Dengzhanxixin injection, a traditional Chinese medicinal plant extensively utilized for ischemic cardio-cerebral vascular diseases, has demonstrated notable efficacy in reducing infarct volume, promoting neuronal cell survival in the ischemic penumbra, and preserving mitochondrial function in brain tissue. These effects, as evidenced through MRI and PET scans, suggest their significant potential to improve neurological and cognitive outcomes in acute ischemic stroke patients (32). Similarly, the combination of Buyang Huanwu Decoction, known for enhancing blood circulation, with olanzapine (33), or NeuroAiD II (MLC901), herbal components known for their neuroprotective and anti-inflammatory properties (34), has shown significant improvements in clinical symptoms, neurological function, and dementia severity in vascular dementia patients’ post-stroke, as evaluated through PET and SPECT scans. This integrative approach indicates superior therapeutic benefits compared to conventional therapy alone, offering promising avenues for managing vascular dementia following cerebral ischemic events.

Meanwhile, mindfulness cognitive therapy has demonstrated significant improvements in cognitive function and emotional well-being among stroke survivors undergoing rehabilitation (53), as evidenced by social-psychological, biochemical, and functional MRI (fMRI) measurements (35). Integration of neuroimaging techniques like fMRI and DTI into rehabilitation programs, such as trunk training using motor imagery, offers valuable insights into neural changes associated with motor recovery and balance function, highlighting neural plasticity and structural reorganization in stroke rehabilitation (36).

##### 3.5.5.2 Predict the post-stroke delirium, neglect, and functional recovery

A significant association exists between spatial neglect and the incidence of delirium in stroke patients. Specifically, decreased connectivity of the basal ganglia and right basal forebrain to the brain stem has been shown to predict severe spatial neglect, which increases the risk of delirium during the acute stroke recovery phase (55). Patients with right hemisphere strokes who exhibit offline anosognosia, or a lack of awareness of their spatial neglect deficits, respond less favorably to rehabilitation targeting spatial awareness and attentional deficits compared to those without anosognosia (64).

Virtual reality (VR) technology has provided a reliable and accurate means of evaluating and training visuospatial neglect, offering a more comprehensive and ecologically valid assessment than traditional paper-and-pencil tests (45). VR-based training interventions have proven effective in enhancing visuospatial awareness and reducing neglect symptoms in patients with stroke and neurological deficits. The immersive and interactive nature of VR environments allows for personalized and engaging training experiences, leading to better rehabilitation outcomes.

Furthermore, non-invasive brain stimulation techniques, such as transcranial magnetic stimulation and transcranial direct current stimulation (tDCS), have shown significant improvements in visuospatial neglect symptoms among chronic neglect stroke patients (47). These interventions target specific neural circuits involved in neglect, promoting neural plasticity and facilitating the restoration of spatial awareness and attentional deficits. The effects of non-invasive brain stimulation have been found to be durable, persisting beyond the intervention period, indicating potential long-term benefits for patients with chronic neglect. Collectively, these findings underscore the potential of non-invasive brain stimulation as an innovative and effective treatment approach for alleviating the debilitating symptoms of chronic neglect and improving stroke patient outcomes.

##### 3.5.5.3 Predict the post-stroke audiovisual defects

Individuals experiencing brain damage (in the temporal and/or frontal areas) or migraine attacks exhibited alterations in auditory processing characterized by increased sensitivity to sound stimuli and heightened perception of loudness (38). The neuropsychological assessments were combined with neurophysiological markers, such as electro-encephalography, magnetoencephalography, and MRI, and the auditory thresholds were found to be significantly lower during migraine attacks compared to the interictal period, indicating enhanced auditory sensitivity during brain damage or migraine episodes. These findings suggest a potential link between temporal or frontal brain damage and migraine pathophysiology and alterations in auditory processing, which points to the need for further research on auditory symptoms in these patients. In this regard, the effectiveness of novel remote rehabilitation interventions, incorporating multiple sensory modalities, in improving visual field impairments in patients with post-stroke brain lesions has been reported (46). In this particular study, participants underwent a tailored telerehabilitation programme involving various sensory stimuli, such as auditory and tactile cues, to stimulate residual vision and facilitate visual field expansion. The results indicate promising outcomes, suggesting that multisensory telerehabilitation holds potential as an effective approach for enhancing visual perception, functional abilities, and the search for neural indicators of improvement in stroke individuals with visual field defects.

##### 3.5.5.4 Predict the post-stroke neural plasticity, aphasia, and functional mobility

PET imaging was utilized to visualize and quantify regional cerebral blood flow and glucose metabolism, mapping functional activation patterns during various motor tasks in chronic stroke patients, revealing elevated blood oxygen-level dependent signals in both lesioned and non-lesioned primary motor cortices during unimanual movements with the affected hand (22). Optimal electrode placement and electrical parameters for MRI-based tDCS have been shown to enhance motor recovery in stroke patients, significantly improving the efficacy of tDCS through precise simulation and positioning (21). Additionally, integrating new wearable technologies, such as a hip walking assist robot (49) and MR-compatible hand-induced robotic systems (50), has led to significant improvements in gait and hand function, stride length, walking speed, and overall stability. fMRI data revealed training-induced neuroplasticity associated with motor control, suggesting neural adaptations and increased cortical activation in response to these rehabilitation interventions. These findings underscore the need for personalized assessments to understand neural reorganization and develop targeted rehabilitation strategies.

##### 3.5.5.5 Predict the post-stroke weakness and spasticity

Sarcopenia, a condition characterized by loss of muscle mass and strength, is prevalent among stroke survivors undergoing rehabilitation (63). Post-stroke individuals with sarcopenia initially showed poorer functional outcomes and slower recovery, emphasizing the need to address sarcopenia in post-stroke rehabilitation to optimize recovery and enhance quality of life. Additionally, detailed mapping of innervation zones within spastic muscles using high-density surface electromyography (EMG) and 3D imaging provided precise localization for guiding botulinum toxin injections and understanding neurodegenerative progression post-stroke (48). Furthermore, the study on opposing needling combined with eye acupuncture therapy in post-stroke hemiplegia patients suggests this approach could be a promising treatment option for reducing muscle spasms and improving outcomes in this population (52). These findings may inform the development of more targeted and effective interventions for individuals with neuromuscular disorders characterized by muscle spasticity.

## 4 Discussion

This review identified various promising applications of nuclear medicine in stroke diagnosis and management. For stroke diagnosis, the best discrimination between stroke and mimics has been observed when utilizing a radiopharmaceutical radiotracer to analyse patients at a high risk of recurrent stroke. For accurate detection of ICH, the use of an Infrascanner, enhanced flat detector CT, Sence device, or Smartcap blood test show high accuracy in distinguishing ischemic from haemorrhagic strokes. The efficacy of MSU with integrated CT and the use of pre-hospital acute stroke care notification helped assess factors crucial for predicting stroke severity assessment, including time of stroke onset, ischemic penumbra volume at risk, and reperfusion success, and might perform better if combined with selected specific brain blood biomarkers. For treatment precision, the incorporation of MRI-based biomarkers, quantifying non-heme iron levels, and MRI-based stroke mechanisms for 4D flow nuclear imaging into scoring systems may identify patients with clinical diffusion mismatches, thereby enabling more precise personalized risk stratification to reduce stroke burden. The integration of radionuclide angiography and a novel PET radiotracer, namely 18F glycoprotein 1, might achieve sufficient sensitivity and specificity to effectively detect thrombi in various anatomical locations across different clinical models. Generally, visualizing and quantifying blood flow speeds to determine the degree of carotid artery stenosis could refine patient selection for stenting or carotid endarterectomy. For stroke prognosis, plasma copeptin and S-100 b protein assay can be added to NIHSS to predict stroke severity and functional outcomes. Through techniques like SPECT and PET with radiotracers of TSPO, co-registration with ferumoxytol-enhanced MRI and CT for functional imaging allows for valuable insights into cerebral perfusion, metabolic activity, neuroinflammatory markers, and receptor binding. To predict post-stroke rehabilitation outcomes, the efficacy of a new wearable hip walking-assist robot and 3D imaging of the botulinum neurotoxin may optimize functional recovery and promote neural plasticity post-stroke.

In the context of the existing literature on nuclear medicine, the novel diagnostic radiotracer ^99*m*^Tc-omberacetam holds promising clinical implications in the management of the hyperacute phase of ischemic stroke (69) by quantifying the α-amino-3-hydroxy-5-methyl-4-isoxazolepropionic acid receptors, which are thought to cause excitotoxicity in brain ischemia. Such quantification not only enhances the accuracy of stroke diagnosis but also provides valuable insights into disease progression and responses to therapy. As research continues to advance, the integration of nuclear medicine into routine clinical practice has the potential to revolutionize stroke management, offer clinicians enhanced diagnostic capabilities, and further improve stroke care.

The application of artificial intelligence (AI) in diagnosing acute stroke using CT and MRI imaging has shown significant advancements and potential. AI systems, particularly those leveraging machine learning and deep learning techniques, offer a level of precision and efficiency that surpasses traditional methods. These AI systems can process and analyze large volumes of imaging data swiftly, identifying subtle patterns and anomalies that may be missed by human reviewers. For instance, convolutional neural networks are particularly effective in image classification tasks, allowing for the automated detection and characterization of stroke-related changes in brain scans with high accuracy (70). Platforms like RapidAI (71) utilize sophisticated algorithms to assist clinicians in making timely and accurate decisions by quickly interpreting CT and MRI scans for suspected stroke cases. These tools not only enhance diagnostic accuracy but also streamline workflow, reduce turnaround times, and facilitate rapid treatment decisions, which are crucial in acute stroke management. The integration of AI in stroke diagnosis thus holds the promise of improving patient outcomes through more efficient and precise imaging analysis, ultimately advancing the field of stroke and neuroradiology.

## 5 Implications for future research

Comparing non-nuclear medicine imaging techniques such as CT and MRI to nuclear medicine imaging in stroke diagnosis reveals distinct advantages and disadvantages. CT and MRI are favored for their rapid acquisition and excellent anatomical detail, essential for quickly identifying hemorrhagic and ischemic strokes. CT excels in acute settings due to its speed, while MRI provides superior soft tissue contrast, making it sensitive to subtle ischemic changes. However, these modalities primarily offer structural insights, potentially missing early functional or metabolic alterations. Conversely, nuclear medicine techniques like PET and SPECT provide invaluable functional and metabolic data, detecting cerebral blood flow and glucose metabolism changes before structural damage becomes apparent. Despite their higher costs, longer acquisition times, and the use of radioactive tracers, nuclear imaging’s ability to highlight early functional disruptions presents a crucial complement to the anatomical clarity of CT and MRI, thereby enriching the diagnostic and management landscape of stroke (72).

The integration of nuclear medicine techniques with traditional imaging modalities represents a significant advancement in the diagnosis and management of stroke. These nuclear methods provide unparalleled insights into cerebral physiology by enabling the visualization of regional blood flow, metabolic activity, and receptor binding in the brain, which are critical for identifying viable but functionally compromised tissue. This functional imaging capability allows for the detection of ischemic penumbra—areas that are at risk but not yet infarcted—thus guiding therapeutic interventions like reperfusion therapy more accurately. When combined with the anatomical precision of CT and MRI, nuclear medicine techniques enhance the diagnostic accuracy and prognostic assessment of stroke. This multimodal approach not only improves the identification of stroke subtypes but also aids in monitoring the effectiveness of treatments, tailoring rehabilitation strategies, and ultimately enhancing patient outcomes. By marrying the anatomical and functional data, clinicians can make more informed decisions, advancing the overall care continuum for stroke patients.

There are shortcomings of nuclear medicine strategies that should be resolved through the introduction of novel radiopharmaceuticals and radiological techniques. Given the multifactorial etiological nature of stroke, the potential to apply nuclear medicine to multimodal neuroimaging paradigms may allow for sufficient sensitivity and specificity to address the heterogeneity of stroke. *De facto* future research should concentrate on nanotechnological technique optimization and standardization in subacute and acute phases of stroke care, together with improvements in clinical activity workflows, to further advance risk stratification, clinical decision making, and personalized diagnosis and management while continuing to consider the unique characteristics of each patient.

## 6 Strengths and limitations

This study provides clinicians and researchers with a detailed and systematic approach to screening and filtering a large number of clinical trials, ensuring the inclusion of high-quality and relevant data. The detailed characterization of trial designs, geographical distribution, and participant demographics enhances the study’s generalizability and relevance across different populations and settings. Furthermore, the rigorous risk of bias assessment provides confidence in the reliability of the findings, while the diverse range of outcome measures and diagnostic techniques examined in the included trials underscores the study’s thoroughness and depth in exploring the multifaceted applications of nuclear medicine in stroke care. Nevertheless, it is imperative to acknowledge that the presented analysis has some limitations, there may be bias in favor of the limited number of clinical trials with interventional measures registered in recent years compared to those conducted in earlier periods. Although the study is primarily focuses on adult populations, future reviews could benefit from a more detailed examination of pediatric stroke trials, despite their rarity, to assess the impact of nuclear medicine on this subgroup, providing insights that could refine diagnostic and therapeutic approaches tailored for younger patients.

The application of nuclear medicine techniques in stroke management is fraught with challenges. These techniques are often expensive and require specialized radiologists and equipment, which limits their widespread use. For instance, not all healthcare facilities have the resources and expertise to perform SPECT or PET scanning. The injection of radioactive materials associated with radiation exposure also poses problems, and the high cost of these imaging procedures can also hinder their routine use and availability. While nuclear medicine techniques can provide a comprehensive view of cerebral function and blood flow, some divergence from the actual pathology of stroke is undeniable. Additional imaging data from other procedures, such as CT or MRI, may be needed to complement the information obtained from nuclear medicine images. As the world grapples with these limitations, demand continues to increase for new radiopharmaceuticals and imaging technologies that are more affordable, convenient, precise, and readily available. Accordingly, a comprehensive approach utilizing multiple imaging techniques is still often required for accurate stroke diagnosis and management.

## 7 Conclusion

This review provides valuable insights into the clinical trials in nuclear medicine that could advance the diagnosis and management of patients with stroke. Although some evidence suggests a potential diagnostic and therapeutic impact of nuclear medicine on defining stroke localization, calculating neurophysiological scores, documenting changes in the late phase of stroke progression, determining responses to thrombolytic therapy, and obtaining information on the presence of sufficient collateral blood supply, conflicting evidence has also highlighted its limited discrimination between stroke and stroke mimics. Rigorous studies are warranted to further integrate nuclear medicine into routine stroke care, with a focus on improving diagnostic and treatment protocols and maximizing stroke patient outcomes.

## Data availability statement

The original contributions presented in the study are included in the article/supplementary material, further inquiries can be directed to the corresponding author.

## Author contributions

HA: Conceptualization, Data curation, Formal analysis, Funding acquisition, Investigation, Methodology, Project administration, Resources, Software, Supervision, Validation, Visualization, Writing–original draft, Writing–review and editing.
